# Functional and Genetic Analysis of Coronavirus Replicase-Transcriptase Proteins

**DOI:** 10.1371/journal.ppat.0010039

**Published:** 2005-12-09

**Authors:** Stanley G Sawicki, Dorothea L Sawicki, Diane Younker, Yvonne Meyer, Volker Thiel, Helen Stokes, Stuart G Siddell

**Affiliations:** 1 Department of Medical Microbiology and Immunology, Medical University of Ohio, Toledo, Ohio, United States of America; 2 Institute of Virology, University of Würzburg, Würzburg, Germany; 3 Department of Cellular and Molecular Medicine, University of Bristol, Bristol, United Kingdom; University of California at San Francisco, United States of America

## Abstract

The coronavirus replicase-transcriptase complex is an assembly of viral and cellular proteins that mediate the synthesis of genome and subgenome-sized mRNAs in the virus-infected cell. Here, we report a genetic and functional analysis of 19 temperature-sensitive *(ts)* mutants of Murine hepatitis virus MHV-A59 that are unable to synthesize viral RNA when the infection is initiated and maintained at the non-permissive temperature. Both classical and biochemical complementation analysis leads us to predict that the majority of MHV-A59 ORF1a replicase gene products (non-structural proteins nsp1–nsp11) form a single complementation group (cistron1) while the replicase gene products encoded in ORF1b (non-structural proteins nsp12–nsp16) are able to function in trans and comprise at least three, and possibly five, further complementation groups (cistrons II–VI). Also, we have identified mutations in the non-structural proteins nsp 4, nsp5, nsp10, nsp12, nsp14, and nsp16 that are responsible for the *ts* phenotype of eight MHV-A59 mutants, which allows us to conclude that these proteins are essential for the assembly of a functional replicase-transcriptase complex. Finally, our analysis of viral RNA synthesis in *ts* mutant virus-infected cells allows us to discriminate three phenotypes with regard to the inability of specific mutants to synthesize viral RNA at the non-permissive temperature. Mutant LA *ts*6 appeared to be defective in continuing negative-strand synthesis, mutant Alb *ts*16 appeared to form negative strands but these were not utilized for positive-strand RNA synthesis, and mutant Alb *ts*22 was defective in the elongation of both positive- and negative-strand RNA. On the basis of these results, we propose a model that describes a pathway for viral RNA synthesis in MHV-A59-infected cells. Further biochemical analysis of these mutants should allow us to identify intermediates in this pathway and elucidate the precise function(s) of the viral replicase proteins involved.

## Synopsis

Coronaviruses infect both humans and animals and are associated mainly with respiratory and enteric diseases. The recent outbreak of SARS emphasizes the need to develop new strategies to control these infections. This paper focuses on the proteins involved in the replication of the coronavirus genome and the production of viral mRNAs in the host cell. These so-called replicase-transcriptase proteins are likely to make good targets for the development of anti-coronaviral drugs. The approach used here is to analyze conditional, temperature-sensitive mutants of Murine hepatitis virus that are normal at 33 °C (the permissive temperature) but are unable to replicate and transcribe viral RNAs at 39.5 °C (the restrictive temperature). By identifying the genetic changes responsible for these temperature-sensitive mutations and by analyzing the precise nature of the defect in RNA synthesis at the restrictive temperature, the authors are able to propose a model that describes a pathway for viral RNA synthesis in the infected cell. Further analysis of these mutants should allow the elucidation of the precise function(s) of the viral proteins involved.

## Introduction

**Figure 1:**
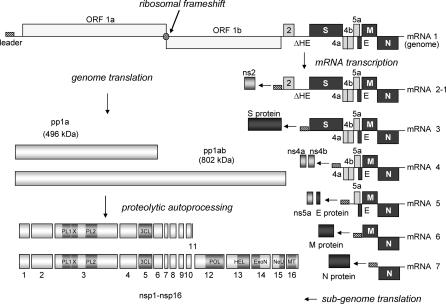
Organization and Expression of the MHV-A59 Genome The structural relationships of the MHV-A59 genome and sub-genomic mRNAs are shown. The virus ORFs are depicted as lightly shaded (replicase proteins), shaded (accessory proteins), and heavily shaded (structural proteins). The ORFs are defined by the genomic sequence of MHV-A59 as published by Coley et al. [45]. The hatched box represents the common 5′ leader sequence and the hatched circle represents the programmed (−1) frameshifting element. The translation products of the genome and sub-genomic mRNAs are depicted and the autoproteolytic processing of the ORF1a and ORF1a/ORF1b polyproteins into non-structural proteins nsp1 to nsp16 is shown. A number of confirmed and putative functional domains in the non-structural proteins are also indicated: 3CL, 3C-like cysteine proteinase; ExoN, exonuclease; HEL, superfamily 1 helicase; MT, S-adenosylmethionine-dependent 2′-*O*-methyl transferase; NeU, endoribonuclease; PL1, papain-like protease 1; PL2, papain-like protease 2; POL, RNA-dependent RNA polymerase; X, adenosine diphosphate-ribose 1′-phosphatase.

Coronaviruses are positive-strand, enveloped RNA viruses that infect vertebrates and are associated mainly with respiratory and enteric disease. They have long been recognized as important pathogens of livestock and companion animals, and they are a common cause of respiratory tract infections in humans [1–3]. More recently, a coronavirus has been identified as the causative agent of SARS, a form of atypical pneumonia in humans with a case fatality ratio of approximately 10% [4]. Clearly, there is an urgent need to develop new strategies to prevent or control coronavirus infections, and understanding the biology, replication, and pathogenesis of these viruses is an essential part of this process. Murine hepatitis virus*,* strain A59 (MHV-A59), is a group II coronavirus with a genome of approximately 31,400 nucleotides. The genomic RNA encodes the structural proteins of the virus, non-structural proteins involved in viral RNA synthesis (the nsp or replicase proteins), and proteins that are non-essential for replication in cell culture but appear to confer a selective advantage in vivo (accessory proteins) [1]. In the MHV-A59-infected cell, the expression of the replicase protein genes is mediated by translation of the genomic RNA, and the expression of the structural protein genes is mediated by the translation of a set of 3′-coterminal subgenomic mRNAs. The subgenomic mRNAs are produced by a unique mechanism that involves discontinuous transcription during negative-strand RNA synthesis [5–7]. The organization and expression of the MHV-A59 genome are illustrated in Figure 1.

The 5′ proximal open reading frames (ORF) of MHV-A59 genomic RNA (ORF1a and ORF1b) are translated to produce two large polyproteins, pp1a and pp1ab, with calculated molecular masses of 496.6 and 802.8 kilodaltons, respectively. Translation of the larger pp1ab involves programmed (−1) ribosomal frameshifting [8]. During or after synthesis, these polypeptides are extensively processed by three virus-encoded proteinases to produce a membrane-bound replicase-transcriptase complex [9]. Cleavage of the replicase polyproteins is predicted to result in 16 end-products; nsp1–nsp11 encoded in ORF1a and nsp12–16 encoded in ORF1b [10]. These proteins have been shown, or are predicted to have multiple enzymatic functions, including papain-like proteases (nsp3), adenosine diphosphate-ribose 1′-phosphatase (nsp3), 3C-like cysteine proteinase (nsp5), RNA-dependent RNA polymerase (nsp12), superfamily 1 helicase (nsp13), exonuclease (nsp14), endoribonuclease (nsp15), and S-adenosylmethionine-dependent 2′-*O*-methyl transferase (nsp16) [11–20]. The crystallographic structures of SARS coronavirus nsp5 and nsp9 have been determined and are likely to be similar for MHV-A59 [21–23].

In the course of an infectious cycle, the MHV-A59 replicase-transcriptase complex amplifies the genomic RNA and synthesizes subgenomic mRNAs. Amplification of the genomic RNA involves full-length negative-strand templates, and the synthesis of subgenomic mRNA involves subgenome-length negative-strand templates [24,25]. The structures engaged in the replication and transcription of positive-strand MHV-A59 RNA have been characterized [26]. Approximately 70% of the replicating and transcribing structures that accumulate in infected cells are multi-stranded intermediates (replicative and transcriptive intermediate RNA, RI/TI RNA) and 30% are found in structures with only one or very few nascent strands (native replicative and transcriptive forms, RF/TF RNA). Although the structures engaged in negative-strand RNA synthesis have not yet been characterized, it is known that MHV negative-strand templates are unstable and turn over during viral replication [27].

The *cis-*acting RNA elements involved in the different phases of MHV RNA synthesis have been studied quite extensively. It has been shown that 5′- and 3′-UTR, as well as 5′-UTR-adjacent regions of the genome are required for MHV replication and transcription [28,29]. Also, studies on MHV, and other nidoviruses, have shown the critical role of the so-called transcription-regulating sequence (TRS) element in the discontinuous phase of the transcription process [7,30–33]. These data show that the stability of the leader-TRS/body-TRS duplex, which forms during the discontinuous extension phase of negative-strand template synthesis, is an important determinant of subgenomic mRNA abundance. However, it is also evident from these studies that the regions flanking the TRS elements have a profound affect on the amounts of subgenomic mRNAs that are produced. In the context of the discontinuous-extension model [5], this is explained as different degrees of “attenuation” at each of the TRS elements during negative-strand synthesis.

In contrast, there is still very little known about the structure, functions, and interactions of viral and cellular proteins in the replicase-transcriptase complex as it is engaged in different modes of RNA synthesis. As mentioned above, bioinformatic and biochemical studies have identified a number of (putative) enzymatic activities associated with individual coronavirus replicase proteins, and a number of cellular proteins have also been implicated as components of the MHV replicase-transcriptase complex [34–36]. However, the essential nature of some of these cellular proteins has been questioned [37], and further work is needed to determine the exact protein composition of the coronavirus replicase-transcriptase complex and how the composition is altered, or how the proteins are modified to regulate the different activities of the complex.

In order to address these sorts of questions, we have embarked upon a detailed analysis of temperature-sensitive *(ts)* mutants of MHV-A59 that are unable to synthesize viral RNA when the infection is initiated and maintained at the non-permissive temperature. The essential feature of these mutants is that they are likely to be defective in different aspects of viral RNA synthesis and a detailed characterization of their genotype and phenotype should provide insights into the mechanisms of RNA synthesis, the functions of individual viral replicase proteins, and the protein-RNA and protein–protein interactions that regulate the activity of the replicase–transcriptase complex. These conditional-lethal mutants may also be used in a *cis*–*trans* test to define the number of complementation groups, or cistrons, that contribute to a specific phenotype. This sort of analysis can also provide valuable insight into the possible pathways that polyproteins must travel to assume functional configurations and has been used with success for other RNA viruses [38].

The MHV-A59 mutants that we study have been produced in a number of laboratories over a period of 20 years [39–41]. They have been selected to have a low efficiency of plaque formation at the non-permissive temperature compared with the permissive temperature and hence a reversion frequency indicative of single point mutations. In this study, we describe a complementation analysis, and by sequence analysis of both *ts* virus and revertants, we identify the causal mutation for eight of these mutants. We also describe a more detailed phenotype for selected mutants and suggest a model that describes the different modes of RNA synthesis during coronavirus replication and transcription.

## Results

### Characterization of *ts* Mutants and Revertants

Table S1 lists the *ts* mutants of MHV-A59 used in our collection. All the *ts* mutants failed to form plaques or synthesize viral RNA when infection was initiated and maintained at the non-permissive temperature. While many mutants failed to form plaques at 37 °C, other mutants formed plaques at 37 °C and were considered leaky. This included Alb *ts*22 that produced pin-prick-sized plaques after 2 d at 37 °C (compared with the wild-type [wt] A59 virus, which produced uniform plaques of 4–5 mm in diameter) and Wü *ts*18, Wü *ts*36, and Wü *ts*38, which produced smaller than wt plaques at 37 °C. However, even for these mutants, the *ts* defects responsible for their RNA-negative phenotype appeared to be caused by a single point mutation because each *ts* mutant possessed a characteristic low reversion frequency between 10^−4^ and 10^−8^ per average base [42]. The virus produced at 37 °C by Alb *ts*22, Wü *ts*18, Wü *ts*36, and Wü *ts*38 was also *ts,* i.e., the efficiency of plating (EOP) was less than 10^−4^.

For most mutants, the revertant virus obtained from plaques formed at the non-permissive temperature had properties identical to wt MHV-A59. One exception was Alb *ts*17, which produced equal numbers of revertant viruses causing A59-sized plaques and revertant viruses with noticeably smaller plaques (Figure S1). We isolated revertant viruses from a large (A59-sized) plaque (Alb 17R_L_) and a small plaque (Alb 17R_S_) for sequence analysis (see below). Some of the *ts* mutants did not produce revertant viruses (e.g., LA *ts*3, Alb *ts*19) or produced revertant viruses that were markedly different from the parental MHV-A59 virus.

### Complementation Analysis

We began our complementation analyses using Alb *ts*16, LA *ts*6, and Alb *ts*22 because they each had a distinct *ts* viral RNA synthesis phenotype (see below). Cells were singly infected or doubly infected with two *ts* mutants and the cells and medium were harvested after the completion of a single round of replication, i.e., 8 h post-infection (hpi) at 40 °C. We also confirmed that if infection with a *ts* virus alone was allowed to proceed for up to 2 h at 30 °C, and then the culture shifted to 40 °C and the virus harvested at 12 hpi, the titer we obtained was low (~10^4^ plaque-forming units [pfu]/ml). Thus, this protocol prevented the production of revertant virus by a second round of replication. Complementation was measured by determining the complementation index (CI) as described in Materials and Methods. By definition, if the mutations are in the same cistron, the viruses will not complement each other. On the other hand, if the mutations are in different cistrons, the mutants will complement each other and progeny *ts* virus will be recovered.

The results of six individual crosses between Alb *ts*16 and LA *ts*6 are shown in Table 1. All of these crosses failed to show complementation. The average CI value was 0.5 (0.5 ± 0.18 SD), which is the theoretical value for two mutants with mutations in the same cistron [43]. This CI value was obtained using only the titers determined at 30 °C and was not corrected for the presence of revertants (or recombinants) as was done by others [39,44]. We found it unnecessary to make this correction because it did not significantly change the CI value (at most a decrease of one tenth) and whether or not the mutants scored as able to complement one another. From these results, we concluded that Alb *ts*16 and LA *ts*6 had a mutation in the same cistron and were, therefore, in the same complementation group. We next determined if Alb *ts*22 would complement Alb *ts*16 or LA *ts*6. As shown in Table 1, in three separate experiments Alb *ts*22 clearly complemented both Alb *ts*16 and LA *ts*6. Therefore, the mutation in Alb *ts*22 was in a different cistron than the mutations in Alb *ts*16 and LA *ts*6, thus identifying a second complementation group. In a series of further experiments, we extended our complementation analysis to include Alb *ts*6, Wü *ts*18, Wü *ts*36, and Wü *ts*38. Using the same assay, we found that Alb *ts*6 complemented Alb *ts*22 but failed to complement Alb *ts*16 or LA *ts*6. Thus, we conclude that Alb *ts*6 was in the same complementation group as Alb *ts*16 and LA *ts*6. Finally, we found that Wü *ts*18, Wü *ts*36, and Wü *ts*38 were in a different complementation group(s) from that of either Alb *ts*6 or Alb *ts*22, and thus, these mutants defined at least a third complementation group.

**Table 1:**
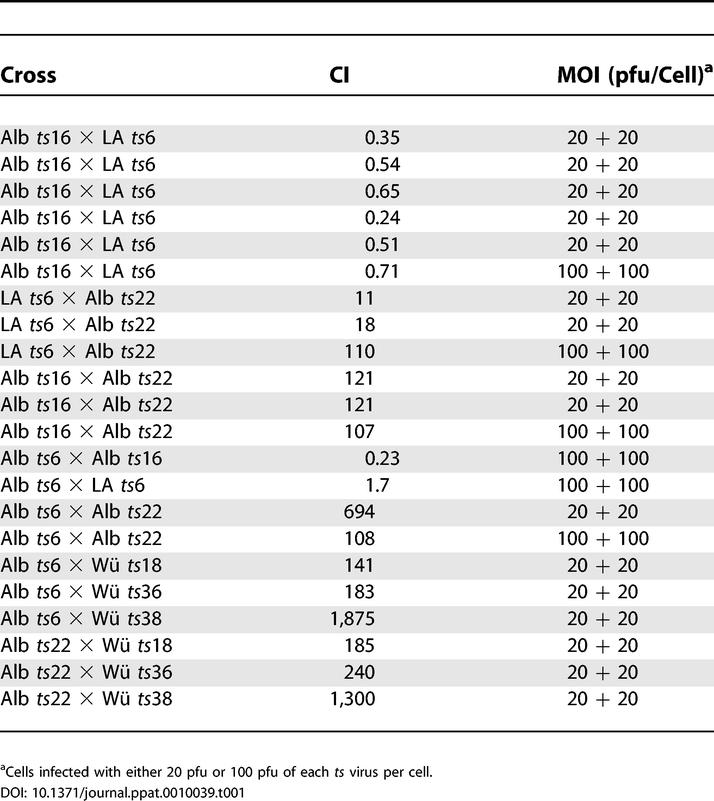
Genetic Complementation Analysis of MHV-A59 *ts* Mutants

In our analysis of the *ts* mutants of MHV-A59 described above, values for the CI were always less than two or more than five and thus readily interpreted as positive or negative without correction for the presence of revertants or recombinants. However, from the results we obtained, it was clear that recombination did occur when there was complementation. The EOP of the virus harvested from cells co-infected with two complementing viruses was usually ~10^−2^, and not the EOP of the individual *ts* mutants, which was 10^−4^–10^−8^. This result is in contrast to similar experiments using Sindbis virus in complementation assays, where we obtained similar EOPs to the input viruses when assaying the progeny from complementing *ts* mutants (unpublished data). We took these results to indicate that complementation allowed recombination in MHV.

**Figure 2:**
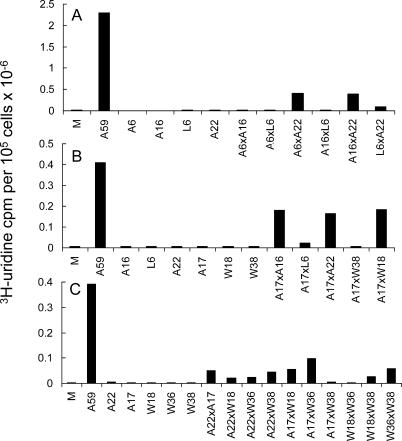
Biochemical Complementation Analysis of Selected MHV-A59 *ts* Mutants Cells were mock-infected or infected with MHV-A59, one of the *ts* mutants, or with a mixture of two *ts* mutants. The cells were incubated at 40 °C in medium containing dactinomycin and ^3^H-uridine and, at 8 hpi, ^3^H-uridine incorporation into trichloroacetic acid-precipitated RNA was determined. Cells were infected with: M, mock-infected; A59, MHV-A59; A6, Alb *ts*6; A16, Alb *ts*16; A22, Alb *ts*22; A17, Alb *ts*17; L6, LA *ts*6; W18, Wü *ts*18; W36, Wü *ts*36; W38, Wü *ts*38; A6xA16, Alb *ts*6 and Alb *ts*16; A6xL6, Alb *ts*6 and LA *ts*6; A6xA22, Alb *ts*6 and Alb *ts*22; A16xL6, Alb *ts*16 and LA *ts*6; A16xA22, Alb *ts*16 and Alb *ts*22; L6xA22, LA *ts*6 and Alb *ts*22; A17x A16, Alb *ts*17 and Alb *ts*16; A17xL6, Alb *ts*17 and LA *ts*6; A17xA22 or A22xA17, Alb *ts*17 and Alb *ts*22; A17xW38, Alb *ts*17 and Wü *ts*38; A17xW18, Alb *ts*17 and Wü *ts*18; A17xW36, Alb *ts*17 and Wü *ts*36; A22xW18, Alb *ts*22 and Wü *ts*18; A22xW36, Alb *ts*22 and Wü *ts*36; A22xW38, Alb *ts*22 and Wü *ts*38; W18xW36, Wü *ts*18 and Wü *ts*36; W18xW38, Wü *ts*18 and Wü *ts*38; W36xW38, Wü *ts*36 and Wü *ts*38.

This finding provided the means to develop a more convenient and more rapid method of determining complementation for MHV-A59 *ts* mutants. We reasoned that because recombination appeared to be driven by complementation, biochemical complementation (i.e., viral RNA synthesis) might be detected in cells co-infected with complementing *ts* mutants, but not in cells infected with *ts* mutants in the same complementation group. We devised such an assay. Cells were infected at the permissive temperature and were then re-fed with medium prewarmed to the non-permissive temperature and containing dactinomycin to inhibit DNA-dependent RNA synthesis and ^3^H-uridine to label viral RNA. The infected cells were incubated until 7–8 hpi at 39 °C to 40 °C or 8–12 hpi at 30 °C, and RNA synthesis was measured by the incorporation of ^3^H-uridine into acid-precipitable material. Figure 2A shows the results of single and double infection with the Alb *ts*6, Alb ts*16*, Alb *ts*22, and LA *ts*6 mutants. The data show that at 40 °C, the mutants Alb *ts*6, Alb *ts*16, and LA *ts*6 were not able to rescue the RNA-negative phenotype of each other and thus, the three mutants were in the same complementation group. In contrast, Alb *ts*22 was able to rescue the RNA-negative phenotype of Alb *ts*6, Alb ts*16,* and LA *ts*6 and thus, was the sole member of a separate complementation group. This result is identical to that obtained using classical complementation assays and served to validate the new method. The assay was as specific as classic genetic complementation, which measures progeny virus production, but is less time-consuming.

Using this assay, we were able not only to confirm the prediction of at least three complementation groups that were obtained using classical complementation procedures but also to identify a fourth complementation group. The results are presented in Figure 2B and 2C and show that mutants Alb *ts*17, Wu *ts*36, Wu *ts*38, and Wu *ts*18 define not one but two additional complementation groups. We found Alb *ts*17 and Wü *ts*38 belong to the same complementation group based on their failure to complement each other's defects. However, both of these mutants complemented Wü *ts*36 and Wü *ts*18, which did not complement each other.

Finally, we extended this assay to include the full collection of mutants that we have available and Table 2 summarizes the complementation patterns of the RNA-negative *ts* mutants of MHV-A59 assayed to date. The numbers shown in Table 2 represent the percentage of viral RNA synthesis found for the mixed mutant-infected cells compared to A59 virus-infected cells at 40 °C. A value less than zero means the ^3^H-uridine incorporation was less than that obtained from mock-infected cells. With this type of assay, we took less than 1% of the MHV-A59 incorporation as indicating failure to complement and greater than 1% as evidence of positive complementation. Based on these results, it was possible to assign a further ten mutants (Alb *ts*2, *ts*8, and *ts*9, and *ts*19; Ut *ts*88 and *ts*329; LA *ts*3 and *ts*9; and NC *ts*2 and *ts*3) to the same complementation group as Alb *ts*6, Alb *ts*16, and LA *ts*6 and, it was possible to assign mutant Ut *ts*145 to the same complementation group as Wü *ts*18 and Wü *ts*36. Thus, it was possible to assign the entire collection of 19 RNA-negative *ts* mutants of MHV to one of four complementation groups, which we have tentatively named cistrons I, II, IV, and VI based on the locations identified for their causal point mutations (see below). This numbering scheme leaves open the possibility of finding two additional complementation groups (cistrons III and V) in the future that would represent gene products of ORF1b (see below).

**Table 2:**
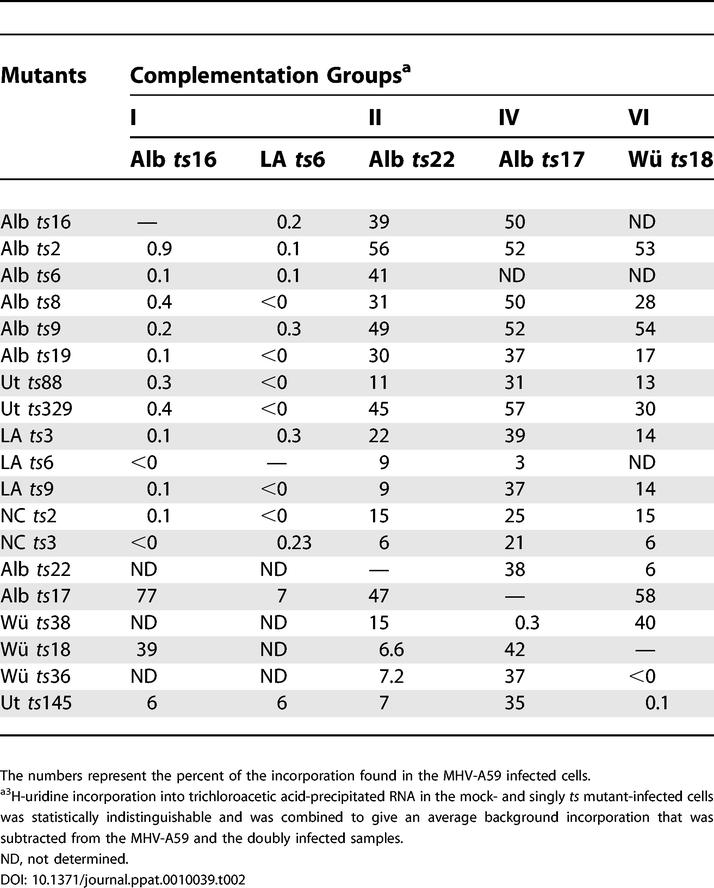
Biochemical Complementation Analysis of MHV-A59 *ts* Mutants

### Identification of Mutations Responsible for the *ts* Mutant Phenotype

**Figure 3:**
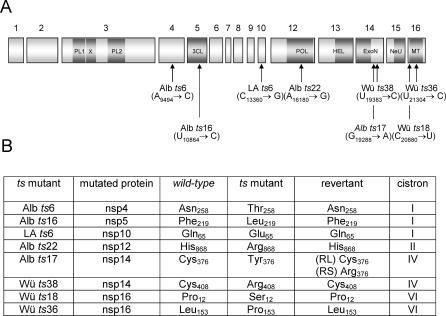
Genotypic Analysis of Selected MHV-A59 *ts* Mutants (A) The positions of mutations responsible for the *ts* phenotype of selected MHV-A59 mutants are illustrated in relation to the non-structural proteins (nsp1–16) produced by proteolytic processing of the ORF1a/ORF1b polyprotein, pp1ab. Nucleotide changes are numbered according to the sequence of the infectious cDNA clone of MHV-A59. (B) The amino acid substitutions responsible for the mutant and revertant phenotypes are listed together with the mutated protein and the cistron to which each mutant has been assigned. The amino acids are numbered from the amino-terminus to the carboxyl-terminus of each of the non-structural proteins.

The entire coding region of the replicase genes (ORF1a and ORF1b) was sequenced for each of eight *ts* mutant/revertant pairs. In each case, a single nucleotide change was identified as the mutation responsible for the *ts* mutant phenotype. Using the numbering that we have assigned to the infectious cDNA clone of the MHV-A59 genome [45] (GenBank accession number AY700211), the nucleotide changes compared to wt MHV-A59 were identified as: Alb *ts*6, A_9494_→ C; Alb *ts*16, U_10864_→ C; LA *ts*6, C_13360_→ G; Alb *ts*22, A_16180_→ G; Alb *ts*17, G_19288_→ A; Wü *ts*38, U_19383_→C; Wü *ts*18, C_20880_→U; Wü *ts*36, U_21304_→ C (Figure 3A). We also identified a number of nucleotide differences between mutants isolated in different laboratories, but in no case did they correlate with the *ts* phenotype. With the exception of the Alb 17R_S_ revertant, all of the revertants we isolated were true, i.e., they were genetically and phenotypically identical to the wt MHV-A59. The Alb 17R_S_ revertant was a pseudorevertant in that the nucleotide at position 19288 had reverted from A→ C, which resulted in a substitution of Tyr with Arg. This radical substitution was reflected in a small plaque phenotype (Figure S1).

All of the nucleotide changes responsible for the *ts* mutant phenotype were non-synonymous mutations. The amino acid substitutions are shown in Figure 3B. Conservative substitutions were identified in nsp5 and nsp10 of the Alb *ts*16 and LA *ts*6 mutants, respectively. Moderately conservative substitutions were identified in nsp4 and nsp12 of the Alb *ts*6 and Alb *ts*22 mutants, respectively. And radical substitutions were identified in nsp14 of the Alb *ts*17 and Wü *ts*38 mutant, as well as nsp16 of the Wü *ts*18 and Wü *ts*36 mutants [46]. A comparison of the predicted replicase protein sequences from different coronaviruses showed that there was, by and large, conservation of the amino acids that were substituted in the proteins with a *ts* phenotype. For example, the Gln_65_ residue of nsp10, the His_868_ residue of nsp12, and the Cys_408_ residue of nsp14 appear to be well conserved in Group I, II (including SARSCoV), and III coronaviruses. In contrast, the Asn_258_ residue of nsp4 is only found in MHV strains, although in the majority of other coronaviruses, it is substituted by an aspartic acid. Finally, it is possible, with different degrees of confidence, to predict the structural environment in which the residues in question are found. On the one hand, it is highly likely that the Phe_219_ residue of nsp5 is located in an extended area that connects the α-helices B and C in the carboxyl-terminal domain III of nsp5, the 3C-like proteinase. This conclusion is based upon the similarity in the sequences of coronavirus nsp5 proteins and the crystallographic structures that have been solved for the transmissible gastroenteritis virus (TGEV), SARSCoV, and HCoV-229E nsp5 proteins [23,47,48]. On the other hand, programs that predict protein secondary structure [49] indicate that the Gln_65_ residue of nsp10, the His_868_ residue of nsp12, and the Cys_408_ residue of nsp14 are located in disordered loop structures, while the Asn_258_ residue of nsp4 and the Leu_153_ residue of nsp16 are involved in α-helices. Obviously, more definitive structural data will be needed to confirm these predictions.

### Phenotypes of the MHV-A59 *ts* Mutants

**Figure 4:**
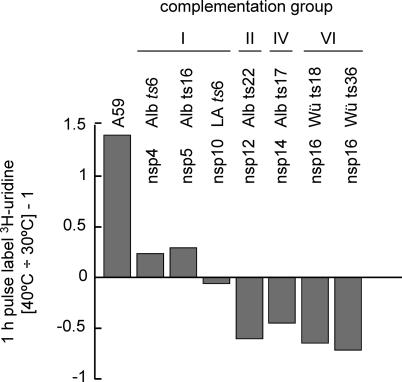
RNA Synthesis Phenotype of MHV-A59 *ts* Mutants RNA synthesis was determined using a 1 h pulse label with ^3^H-uridine in the presence of dactinomycin and cycloheximide, given to wt MHV-A59 and *ts* mutant virus-infected cells at 8 hpi with or without shifting from the permissive to the non-permissive temperature. The amount of incorporated ^3^H-uridine at 40 °C was divided by that at 30 °C and 1.0 was subtracted. The results represent the average of five separate experiments. A value of zero means the incorporation at the two temperatures was the same.

We focused our phenotypic analysis on the eight MHV-A59 *ts* mutants that had been genotyped and began by measuring “total” viral RNA synthesis in infected cells prior to and following shift from the permissive to the non-permissive temperature. This analysis was done after 8 h of incubation at 30 °C, a time at which the replicase-transciptase complex produces mainly (>90%) positive-strand RNA, and ~20% of the maximum rate of RNA synthesis has been reached. Mutant virus-infected cells were shifted to 40 °C at 8 hpi and a duplicate set was left at 30 °C. Both sets of cultures were labeled for 1 h with ^3^H-uridine in the presence of 10 μg per ml of cycloheximide (CH) to monitor the replicase-transcriptase activity at the time of shift. The results are shown in Figure 4. In MHV-A59 infected cells, the amount of ^3^H-uridine incorporation doubled, as expected, when the temperature was increased by 10 °C. The group I mutants had about the same level of viral RNA synthesis at both temperatures, while in the group II, IV, and VI mutant-infected cells, viral RNA synthesis diminished by 50% or more in the hour following temperature shift. We interpret this to mean that mutations in replicase proteins encoded in ORF1a appeared to confer temperature-sensitivity to the viral replicase-transcriptase complex, but once it had formed at 30 °C, its positive-strand synthetic activity was relatively resistant to higher temperature. In contrast, mutations in ORF1b-encoded proteins, namely nsp12, nsp14, and nsp16 appeared to affect the positive-strand synthetic activity of already-formed replicase-transcriptase complexes. We then went on to analyze the phenotypes of three *ts* mutants in more detail.

#### Alb *ts*22.

**Figure 5:**
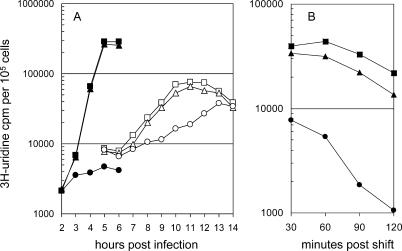
RNA Synthesis Phenotype of the Alb *ts*22 Mutant RNA synthesis was determined (A) using 1 h pulse labels with ^3^H-uridine in the presence of dactinomycin, given to MHV-A59-, Alb *ts*22-, and Alb 22R-infected cells 1–6 hpi at 40 °C or 5–14 hpi at 30 °C; █, 40 °C, wt MHV-A59; ▴, 40 °C, Alb 22R; •, 40 °C, Alb *ts*22; □, 30 °C, wt MHV-A59; ▵, 30 °C, Alb 22R; ○, 30 °C, Alb *ts*22, or (B) using 30 min pulse labels with ^3^H-uridine in the presence of dactinomycin, given to MHV-A59-, Alb *ts*22-, and Alb 22R-infected cells after shift from the permissive to the non-permissive temperature at 13 hpi; █, wt MHV-A59; ▴, Alb 22R; •, Alb *ts*22.

The phenotype described above for group II, IV, and VI mutants would be consistent with a defect in any stage of positive-strand RNA synthesis. In the case of mutant Alb *ts*22, however, we have shown that the *ts* lesion is located in nsp12, the viral RNA-dependent RNA polymerase subunit. This suggested to us that the Alb *ts*22 might be defective in the elongation phase of RNA synthesis. To analyze the phenotype of Alb *ts*22 in more detail, RNA synthesis in Alb *ts*22-infected cells was determined using 1 h pulse labels with ^3^H-uridine in the presence of dactinomycin, given between 1–6 hpi at 40 °C or between 5–14 hpi at 30 °C (Figure 5A). At 40 °C, Alb *ts*22-infected cells incorporated only mock levels of ^3^H-uridine, as expected for an RNA-negative *ts* mutant. In contrast, cells infected with wt MHV-A59 or with Alb 22R (a revertant of Alb *ts*22) made RNA at high rates and at identical times. At 30 °C, Alb *ts*22 was defective in viral RNA synthesis and never reached the levels of viral RNA synthesis shown by wt MHV-A59 or Alb 22R. These results are consistent with our finding that, at 30 °C, the plaques formed by Alb *ts*22 were smaller that those formed by wt MHV-A59. Analysis by gel electrophoresis of the species of positive-strand RNA made in Alb *ts*22-infected cells at 30 °C showed the typical pattern of seven RNAs (genome and six subgenomic mRNAs), although the six subgenomic mRNAs were reduced equally in amount relative to the genome RNA when compared to Alb 22R infected cells (unpublished data). We conclude that Alb *ts*22 not only produced less overall RNA compared to wt MHV-A59 and Alb 22R, even at the permissive temperature, but also under-produced all of the subgenomic mRNA species relative to the genome RNA.

We also examined the ability of Alb *ts*22-infected cells to continue viral RNA synthesis after shift from 30 °C to 40 °C at 13 hpi (Figure 5B). This allowed us to follow the activity at 40 °C of the viral RNA-dependent RNA polymerase that was made and assembled at 30 °C. At this time, Alb *ts*22 RNA synthesis was at its maximum rate and RNA synthesis by wt MHV-A59 and Alb 22R was declining. The results show that a shift to 40 °C led to the rapid loss of RNA synthesis by Alb *ts*22 but not by wt MHV-A59 or Alb 22R. This result is consistent with a failure of the viral RNA-dependent RNA polymerase to continue transcription at the non-permissive temperature. We concluded Alb *ts*22 had a *ts* defect in elongation, although we do not know if elongation is directly affected or if the amino acid change in nsp12 affects its interaction with an as yet unknown, but essential protein. We have also shown that, as expected, Alb *ts*22 is unable to synthesize negative-strand RNA at the non-permissive temperature (unpublished data).

#### Alb *ts*16 and LA *ts*6.

**Figure 6:**
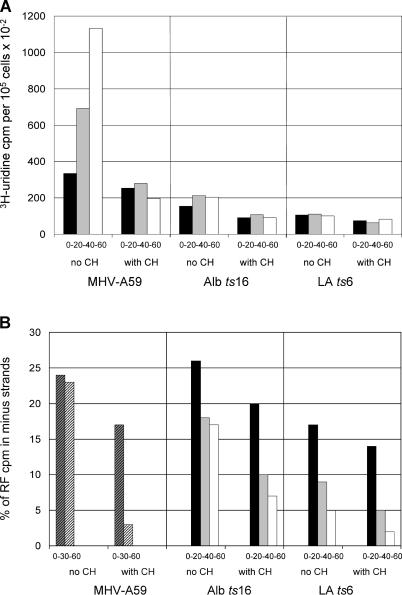
RNA Synthesis Phenotype of the Alb *ts*16 and LA *ts*6 Mutants RNA synthesis (A) or negative-strand RNA synthesis (B) was determined using 20 or 30 min pulse labels with ^3^H-uridine in the presence of dactinomycin, with or without the addition of CH, after shifting the incubation temperature of MHV-A59-, Alb *ts*16-, and LA *ts*6-infected cells from 30 °C to 40 °C at 8 hpi: filled bar, 0–20 min pulse; grey bar, 20–40 min pulse; open bar, 40–60 min pulse; dark diagonal bar, 0–30 min pulse; light diagonal bar, 30–60 min pulse.

Although both Alb *ts*16 and LA ts6 are unable to synthesize viral RNA when the infection is initiated and maintained at the non-permissive temperature, the data shown in Figure 4 suggests that they are not significantly impaired in their ability to synthesize positive-strand RNA at this temperature. This conclusion is strengthened by the results shown in Figure 6A, which demonstrate the kinetics of overall viral RNA synthesis in Alb *ts*16 and LA *ts*6 virus-infected cells after shifting the incubation temperature from 30 °C to 40 °C at 8 hpi. With wt MHV-A59, viral RNA synthesis increased rapidly within the first 60 min after temperature shift, consistent with the synthesis of both additional negative-strand templates and their nascent positive-strand product. The addition of CH at the time of shift resulted in a constant rate of viral RNA synthesis for at least 1 h. As we know that negative-strand synthesis in MHV-A59-infected cells is short-lived and stops within 30 min of the inhibition of protein synthesis [24], we deduce that the addition of CH prevented the synthesis of new viral proteins, which in turn prevented the formation of additional replicase-transcriptase activity and negative-strand templates.

In cells infected with complementation group I *ts* mutants Alb *ts*16 and LA *ts*6, viral RNA synthesis continued at 40 °C at the level ongoing at the time of temperature shift (Figure 6A). This meant that the replicase-transcriptase complexes assembled at 30 °C continued to function at 40 °C in the synthesis of positive-strand RNA. However, unlike A59-infected cells, the group I mutants did not increase their rates of RNA synthesis after shift to non-permissive temperature, indicating that no new active complexes were formed. This phenotype resembled that seen with MHV-A59-infected cells treated with CH, and we conclude that the complementation group I mutants are defective in their ability to form active replicase-transcriptase complexes at 40 °C but retain the positive-strand synthesis activity of the complexes formed at the permissive temperature.

At least two possibilities could account for a failure of group I *ts* mutants to form fully competent replicase-transcriptase complexes at the non-permissive temperature. Either no new negative-strand templates were made, i.e., a defect in negative-strand synthesis, or, if they were made, they could not be used as templates for positive-strand synthesis. The latter phenotype has been observed for certain alphavirus mutants [50], which were called conversion-defective mutants. To distinguish between these two possibilities, it is necessary to shift the *ts* mutant-infected cells to the non-permissive temperature and determine their ability to continue negative-strand RNA synthesis. Mutants that fail to continue negative-strand RNA synthesis would be defective in this step, while mutants that continued to make negative strands would be designated as conversion-defective mutants. Cells infected with wt MHV-A59, Alb *ts*16, and LA *ts*6 were shifted from 30 °C to 40 °C at 8 h (Alb *ts*16) or 9 h (LA *ts*6) post-infection and were pulse-labeled with ^3^H uridine at 40 °C. Then, viral negative-strand templates in replicating and transcribing structures were purified free of single-stranded RNA, and the incorporation of radioactivity into negative-stranded RNA was measured by nuclease protection assays. In this assay, the results are expressed as the percentage of the ^3^H-uridine incorporated into the negative-stranded component of the purified, nuclease-resistant RNA cores of the replicative-transcriptive structures. As these structures represent double-stranded RNA, if 40%–50% of the total incorporation in the core RNA is found in negative strands, it means that 80%–100% of the negative strands that were active as templates during the pulse period had been made during this same period. This occurred when negative-strand synthesis was measured early in the infection cycle, when viral RNA synthesis was ~20% of the maximum [24].

Figure 6B shows that in wt MHV-A59-infected cells, negative-strand synthesis continues following a shift from 30 °C to 40 °C at a time when the amount of viral RNA synthesis is ~20% of maximum. This is seen by the similar high values of 20%–25% of the labeled RF RNA being in negative strands for successive 30 min pulse-periods in the absence of CH. Also, as shown previously, continued negative-strand synthesis in MHV-A59-infected cells is dependent on continued translation and abruptly declines in the presence of CH. In the case of LA *ts*6, the percentage of ^3^H-uridine incorporated in negative strands declined abruptly after shifting to 40 °C and this decline was the same in the absence or the presence of CH. With Alb *ts*16, negative-strand synthesis continued during the 20–40 min and the 40–60 min pulse-periods in the absence of CH but was inhibited in the presence of CH. For this mutant, to find that negative-strand synthesis continued at 40 °C without an increase in the rate of positive- strand synthesis, as seen for MHV-A59, was consistent with Alb *ts*16 having a *ts* defect affecting the ability of the negative-strand templates to be efficiently used at 40 °C. Thus, we conclude that LA *ts*6 was defective in continuing negative-strand synthesis after shift to 40 °C and Alb *ts*16 displayed what appears to be a conversion phenotype.

## Discussion

Taken together with the complementation analysis, the identification of the mutations responsible for the *ts* phenotypes of Alb *ts*6, Alb *ts*16, Alb *ts*17, Alb *ts*22, LA *ts*6, Wü *ts*18, Wü *ts*36, and Wü *ts*38 leads to a number of important conclusions. First, our data strongly suggest that most of the replicase gene products of ORF1a are *cis-*active and form a single complementation group (cistron I) encompassing, at least, the nsp4 to nsp10 coding region. If correct, our conclusion must mean that a large proportion of nsp1–nsp11 proteins function as a polyprotein, if only initially or transiently, or they associate as a *cis-*acting complex before they are proteolytically processed. We favor a model in which a pp1a-related polyprotein represents a large modular scaffolding protein that displays binding sites for ORF1b-encoded pp1ab processing products. While the large number of mutants that fall into cistron I clearly suggest that it is extensive and polygenic, it is not yet clear if all of the ORF1a-encoded proteins function in *cis.* We are aware that the arterivirus Equine arteritis virus ORF1a-encoded protein nsp1 can function in *trans* [51] and it has recently been shown that the MHV-A59 ORF1a-encoded protein nsp2 is non-essential for virus replication [52]. The genetic analysis of further MHV-A59 *ts* mutants will be needed to define the precise boundaries of MHV-A59 cistron I.

Second, our results suggest that the replicase gene products encoded in ORF1b (i.e., nsp12–nsp16) are diffusible and thus assemble and function in viral RNA synthesis after cleavage from pp1ab. This also leads us to the prediction that there will be five cistrons in ORF1b, each corresponding to one of the proteolytic cleavage products, and we have designated them tentatively as cistrons II–VI in a 5′ to 3′ direction (nsp12, cistron II; nsp13, cistron III; nsp14, cistron IV; nsp15, cistron V; and nsp16, cistron VI). The idea that the MHV-A59 ORF1b-encoded replicase proteins function in trans is consistent with the results of Brockway et al., who have shown that a green fluorescent protein–tagged MHV-A59 nsp12 is able to diffuse into the replicase-transcriptase complex if expressed individually in virus-infected cells [9]. However, we would also like to note that our data does not exclude the possibility that some of the ORF1b-encoded proteins may function as intermediates, rather than the end products of proteolytic cleavage. For example, functional proteins comprising nsp12/nsp13, nsp13/nsp14, nsp14/nsp15, nsp15/nsp16 as well as nsp13/nsp14/nsp15 could all be accommodated as single cistrons based upon our complementation data. This would lead to the prediction of either three or four cistrons encoded in ORF1b. The idea that a number of the enzymes involved in coronavirus RNA synthesis may be linked not only functionally, i.e., sequentially in a concerted reaction pathway, but also structurally (i.e., as multifunctional proteins) is also suggested by other studies. For example, Ziebuhr and colleagues [53] have shown that 2′-*O*-ribose-methylated RNA substrates are resistant to cleavage by the SARS-coronavirus endoribonuclease (nsp15), indicating a functional link with the S-adenosylmethionine-dependent 2′-*O*-methyl transferase (nsp16). We are currently searching for further *ts* mutants that might help resolve this issue and we are attempting to *trans*-complement *ts* mutants with cell lines that constitutively express ORF1b-encoded replicase proteins. Despite these reservations, the genetic data do allow us to conclude that not only nsp5, the 3C-like cysteine proteinase, and nsp12, the putative RNA-dependent polymerase (as might have been predicted), but also nsp14, the putative MHV exonuclease, nsp16, the putative MHV 2′-*O*-methyltransferase, nsp4, and nsp10 are essential for the assembly of a functional replicase-transcriptase complex.

In contrast to most other positive-stranded RNA virus, the viral replicase-transcriptase complex of coronaviruses (and most other nidoviruses) functions to amplify the genome via a full-length negative-strand intermediate and to produce, via a discontinuous process, subgenome-length negative-strand templates that are then copied directly into subgenomic mRNA. How the replicase-transcriptase complex accomplishes these various activities is not understood in any detail. For example, it is not known whether the same replicase-transcriptase complex functions to produce full-length and subgenome-length RNA or how the conversion from negative- to positive-strand RNA synthesis is regulated. Does the analysis of MHV-A59 *ts* mutants help us to understand these complex processes?

We have shown previously that negative- and positive-strand RNA synthesis occurs simultaneously throughout MHV-A59 infection but that negative-strand synthesis is short-lived, i.e., its synthesis halts within several minutes after protein synthesis is inhibited [24]. This contrasts with positive-strand synthesis, which continues unabated for 1 h and then gradually declines and disappears about 4 h after the inhibition of translation. These observations suggest that unprocessed forms of the replicase polyprotein(s) might function in negative-strand synthesis and that cleavage of the nascent polyprotein inactivates the negative-strand activity of the replicase, as it does for alphaviruses [54,55]. The replicase-transcriptase activity for positive-strand synthesis can be restarted after the block of translation is reversed [27] but, for this to happen, new negative-strand templates need to be synthesized. In other words, it appears that the coronavirus replicase-transcriptase complex ages, losing both its negative-strand templates and its activity. This interpretation fits well with our genetic analysis of the mutants LA *ts*6, Alb *ts*16, and Alb *ts*6, which shows that they all fall into a single complementation group. It is also consistent with our proposal that the replicase proteins encoded in ORF1a are expressed and function as a polyprotein, or that they assemble as a *cis*-acting complex before they are proteolytically processed. It is also worth noting that in vivo protein labeling experiments indicate that proteolytic processing of both MHV-A59 ORF1a and MHV-A59 ORF1b-encoded replicase proteins is measured in hours rather than minutes [56–58] and that the fully processed 3C-like cysteine proteinase is first detected several hours post-infection [59], a time at which the rate of viral RNA synthesis is already increasing rapidly [24].

The idea that the MHV replicase-transcriptase complex is active in negative-strand RNA synthesis before pp1a is extensively processed also fits well with our detailed phenotypic analysis of cistron I mutants. In the case of LA *ts*6, negative-strand synthesis was inhibited after shift to the non-permissive temperature and, in time, this leads to a decline in positive-strand RNA synthesis (unpublished data). Thus, at the non-permissive temperature, LA *ts*6 could not sustain positive-strand synthesis, nor replace or replenish aging replicase-transcriptase complexes. The causal mutation in LA *ts*6 would substitute a Glu for the Gln_65_ residue of wt nsp10. As noted above the Gln_65_ residue is conserved in Group I, II (including SARSCoV), and III coronaviruses and its substitution with Glu might prevent the proper folding of pp1a into a conformation that would allow it to participate in the formation of a replicase-transcriptase complex with negative-strand activity. It would be interesting to determine if, at the non-permissive temperature, nsp10 of LA *ts*6 could associate with nsp12, nsp13, nsp14, nsp15, or nsp16. Also, it was curious that LA *ts*6 had a very low reversion frequency of ~10^−8^. Why certain bases fail to revert at the typical frequency of 10^−4^ to 10^−5^ is unknown but may be indicative of a region of the genome that is transcribed with higher fidelity than other regions. Alternatively, this low reversion frequency may be an inherent property of the LA *ts*6 replicase-transcriptase complex.

In contrast to LA *ts*6, Alb *ts*16 appeared to be able to continue to form negative strands after shift to the non-permissive temperature, but these negative strands were not converted into templates for positive-strand synthesis. We speculate that Alb *ts16* has a *ts* defect in the conversion of the replicase-transcriptase complex from one able to synthesize negative strands to one able to synthesize positive strands. It is certainly suggestive that Alb *ts*16 had a mutation in nsp5, which is the 3C-like proteinase of the virus, but it has yet to be determined if this mutation affects the activity of the proteinase, or if it affects the folding of pp1a or pp1ab, or if the nsp5 C-terminal domain itself could have a function in positive-strand RNA synthesis. Nevertheless, because negative-strand RNA synthesis was inhibited in Alb *ts*16-infected cells treated with CH at the time of shift to non-permissive temperature, we propose that the Alb *ts*16 replicase-transcriptase complex does not retain its activity for minus-strand synthesis. Rather it fails to gain positive-strand synthesis activity at the non-permissive temperature. We favor a model where the activity that makes positive strands is gained at the expense or loss of the activity to make negative strands.

Finally, although we are able to rationalize the genotype of Alb *ts*22, i.e., a mutation in nsp12 (the RNA dependent RNA polymerase) with its phenotype (i.e., an immediate effect on RNA synthesis at the non-permissive temperature) we were surprised to find that Alb *ts*17, Wü *ts*18, Wü *ts*36, and Wü *ts*38 also showed the same phenotype but had mutations in other replicase proteins. Generally, it is unusual to find so many *ts* mutants that show an effect on RNA synthesis if the replicase-transcriptase complex is first allowed to assemble at the permissive temperature. Most RNA-negative *ts* mutants of alphaviruses, for example, fail to make viral RNA when the infection is initiated at the non-permissive temperature but continue to make viral RNA if shifted to the non-permissive temperature late in infection (unpublished data). One possibility is that nsp14 and nsp16 dissociate or become less tightly associated with the replicase-transcriptase complex after shifting to the non-permissive temperature and this causes the complex to lose elongation activity. Another possibility is that the enzymatic activities associated with nsp14 and nsp16 are altered in the group IV and group VI mutants. Further studies will be required to explain this phenotype.

**Figure 7:**
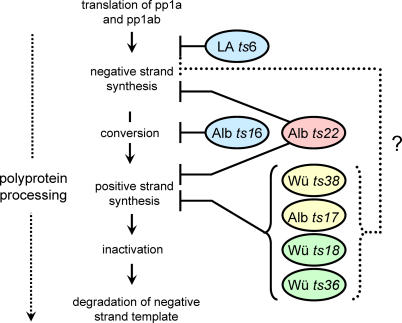
A Model to Describe the Pathway for Viral RNA Synthesis in MHV-A59-Infected Cells Shows a working model that describes a pathway for viral RNA synthesis in MHV-A59-infected cells. The model proposes that the replicase-transcriptase complex forms initially and creates a negative-strand template. It is then converted to utilize the negative strand as a template for positive-strand synthesis and, finally, the complex is inactivated by the degradation of negative-strand templates. It is also proposed that proteolytic processing of the replicase polyproteins plays a role in regulation of this pathway. Also depicted are the putative defects of specific MHV-A59 *ts* mutants. It remains to be shown whether or not the group IV and VI mutants (Wü *ts*38, Alb *ts*17, Wü *ts*18, and Wü *ts*36) are defective in negative-strand RNA synthesis at the non-permissive temperature.

In summary, our detailed phenotypic analysis of MHV-A59 *ts* mutants allows us to propose a working model that describes a pathway for viral RNA synthesis in MHV-A59-infected cells. In this model, the replicase-transcriptase complex forms initially and creates a negative-strand template. It is then converted to utilize the negative strand as a template for positive-strand synthesis and, finally, the complex is inactivated by the degradation of negative-strand templates (Figure 7). Our analysis also allows us to place some of our *ts* mutants at specific points on this pathway. We hope that a more detailed biochemical analysis of these mutants will allow us to identify intermediates in the pathway of RNA synthesis and will provide valuable information of the precise function(s) of the viral replicase proteins involved. Furthermore, we believe that the characterization of these mutants provides an excellent starting point for the generation of second site reversion mutants. This could be done, for example, by using the recently developed MHV reverse genetic system [45] to generate *ts* mutants with codon, rather than nucleotide substitutions. Second site reversion mutants may then provide valuable information on protein-protein interactions within the replicase-transcriptase complex.

## Materials and Methods

### Cells and viruses.

Seventeen clone one (17Cl-1) mouse fibroblast cells [60] were cultured at 37 °C in Dulbecco's modified Eagle's medium (DMEM) supplemented with 6% fetal bovine serum (FBS), 5% tryptose phosphate broth (TPB), penicillin (100 units/ml), and streptomycin (100 μg/ml). Sac(−) cells [61] were cultured at 37 °C in minimal essential medium (MEM) supplemented with 5% FBS, penicillin (100 units/ml), and streptomycin (100 μg/ml). The A59 strain of MHV and a set of *ts* mutants derived from MHV-A59 (Alb prefix) were originally obtained from the laboratory of L. Sturman, Wadsworth Center for Laboratories and Research, Albany, New York, United States [40]. Mutants prefixed with LA (Los Angeles) and NC (North Carolina) were obtained from M. Schaad and R. Baric, University of North Carolina, Chapel Hill, North Carolina, United States and have been initially characterized [39]. Mutants prefixed with Ut (Utrecht) were obtained from W. Spaan, Leiden University Medical Center, Leiden, The Netherlands and have been initially characterized [41]. The LA, NC, and Ut *ts* mutants were derived from different but related lineages of the Albany isolate of MHV-A59. Mutants prefixed with Wü (Würzburg, Germany) were isolated as described below.

For our studies, virus stocks were derived from the original mutant isolates after plaque purification and propagation in 17Cl-1 cells cultured at 30 °C or 33 °C in low pH DMEM (pH 6.4) containing 6% FBS, 5% TPB, penicillin (100 units/ml), and streptomycin (100 μg/ml) [24]. Revertants were picked from plaques of mutants titered at 39.5 °C, followed by another plaque purification at 39.5 °C. The virus stocks used were first passage and were obtained by using virus from a single plaque (~10^7^ pfu) to infect a dish of ~4 × 10^7^ 17Cl-1 cells to yield 30 ml of stock virus with a titer of 1–6 × 10^9^ pfu/ml.

### Isolation of Wü *ts* mutants.

Sac(−) cells were infected with 10 pfu/cell of MHV-A59 (originally obtained from P. Carthew, Medical Research Council Laboratories, Carshalton, United Kingdom), and incubated for 15 h at 37 °C in medium containing 150 μg/ml of 5-fluorouracil. This concentration of pyrimidine analogue was determined to inhibit virus replication by 80%. The mutagenized virus stock was diluted to 15 pfu/ml in medium and 100 μl aliquots were incubated with 10^4^ Sac(−) cells at 30 °C for 48 h. The supernatant was taken from cultures that displayed syncytium formation and used to infect duplicate cultures of 10^4^ Sac(−) cells that were incubated at 30 °C or 39.5 °C for 24 h. The supernatant was taken from replica cultures that developed cytopathic effect at 30 °C but not at 39.5 °C, and potential *ts* mutants were isolated by two plaque purifications. Sequence analysis of the Würzburg strain of MHV-A59 suggests that approximately 8,000 nucleotides at the 5′ end of the genome have been exchanged by recombination with a related but different MHV strain (unpublished data).

### Characterization of mutant stocks for titer and EOP.

17Cl-1 cells in 60 mm petri dishes were infected with 0.5 ml of 10-fold dilutions of virus at room temperature. Virus was diluted in an infection medium (MEM with Hank's balanced salts [HBSS] containing 50 μg/ml of DEAE-dextran [diethylaminoethyl dextran], 0.2% bovine serum albumin, and 20 mM HEPES [N-2-hydroxyethylpiperazine-N′-2-ethansulfonic acid] [pH 6.6]). The inoculum was removed after 30 min and the cells were overlaid with DMEM containing 6% FBS, penicillin (100 units/ml), streptomycin (100 μg/ml), and 0.1% Gelrite™ gellan gum (Sigma, St. Louis, Missouri, United States) and incubated at the appropriate temperature in 7.5% CO_2_. After incubation for 3 or 4 d at 30 °C or 2 d at 37 °C or 39.5 °C, cells were rinsed with 0.15 M NaCl, fixed with methanol, and stained with a solution of 0.2% toluidine blue, 0.2% azure blue, and 1% boric acid. The EOP was calculated by dividing titers at 39.5 °C by the titer at 30 °C. We found that all of the *ts* mutants produced the same titer at 30 °C as at 33 °C and in all cases ± 39 °C was non-permissive for plaque formation of the *ts* mutants.

### Complementation analysis.

Classic or genetic complementation was done by infecting 17Cl-1cells in 35 mm petri dishes either singly with each mutant or doubly with two mutants at a multiplicity of infection (MOI) of 20–30 pfu/cell. After incubation at room temperature or 30 °C for 30 min, the virus inoculum was removed and the infected cells were rinsed with HBSS and re-fed with low pH DMEM or DMEM supplemented with 6% FBS, penicillin (100 units/ml), and streptomycin (100g/ml). The infected cells were incubated at 39.5 °C or 40 °C in 10% CO_2_. One hour later the cells were rinsed again and re-fed with warmed medium and the dishes returned to the incubator until 8 hpi. The medium was harvested, clarified at 10,000 rpm for 5 min, and virus titers were determined by plaque assays at 30 °C. CIs were calculated using the following formula:


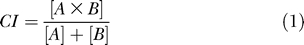


A CI greater than two between mutant pairs was consistent with complementation, i.e., ± 4-fold difference above background, while a CI less than two was negative for complementation [43].

Biochemical complementation was done by mock-infecting or infecting 17Cl-1cells at 30 pfu/cell with MHV-A59, or one of the *ts* mutants, or with a mix of 15 pfu/cell each of two *ts* mutants. After the adsorption period at room temperature for 30 min, the virus inoculum was removed, 1 ml of prewarmed medium containing dactinomycin and ^3^H-uridine (1.85 MBq/ml) was added and the cells were incubated at 40 °C. The wt and the *ts* mutant-infected cells were harvested at 8 hpi and the ^3^H-uridine incorporation into trichloroacetic acid-precipitated RNA was determined.

### Viral RNA synthesis.

Viral RNA synthesis was measured by determining the amount of ^3^H-uridine incorporated in the presence of dactinomycin (20 μg/ml) into acid-precipitable material. [5-^3^H] uridine (≥1.0 TBq/mmol) was added to the medium at either 1.85 or 7.4 MBq/ml. After incubation, the radioactive medium was removed and the cells dissolved with 5% lithium dodecyl sulfate and 200 μg/ml proteinase K in LEH buffer (0.1 M LiCl, 0.001 M EDTA, 0.01 M HEPES, [pH 6.6]) at 2–5 × 10^5^ cells per ml. The DNA was sheared by repeated passage through a 27-gauge needle attached to a 1-ml tuberculin syringe. Triplicate samples of 5 × 10^4^ cells were precipitated with trichloroacetic acid and the precipitates collected on glass fiber filters (Whatman Incorporated, Clifton, New Jersey, United States), dried under a heat lamp, and the radioactivity determined by liquid scintillation spectroscopy. To measure negative-strand synthesis, the dissolved cells were extracted with low pH phenol (pH 4.3), which removed DNA from the aqueous phase, and then with cholorofom:isoamyl alcohol (95:5), and the RNA was precipitated with ethanol. RF RNA was generated by treatment of the RNA with RNase T1 (1U/ug RNA, 30 °C for 30 min in 0.3 M NaCl) and collected by chromatography on CF-11 cellulose and ethanol precipitation. The incorporation of ^3^H-uridine into negative strands was measured by denaturing the RF RNA with heat and annealing in the presence of >100-fold excess of unlabeled RNA obtained from purified virions of MHV [24].

### Isolation of viral RNA.

Two different procedures were used to obtain viral RNA for RT-PCR and sequencing. Virions were purified from ~3 × 10^8^ 17Cl-1 cells that had been infected at a MOI of ~10 pfu/cell and incubated in low pH DMEM at 30–33 °C. The medium from the infected cells (~225 ml) was collected at 24 hpi and clarified at 4,000 rpm for 30 min. The virions were pelleted by centrifugation at 24,000 rpm for 3 h at 4 °C. The virus pellet was allowed to suspend overnight on ice in 0.4 ml/tube of 0.15M NaCl and 20 mM HEPES (pH 6.6). The suspended virus from six tubes was pooled and layered over one SW28 tube containing a linear gradient of 40% potassium tartrate (bottom) and 20% glycerol (top), in 0.0.2 M HEPES (pH 7.4). After centrifugation at 24,000 rpm for 3–4 h at 4 °C, the visible band containing the virions was collected, diluted, and pelleted by centrifugation at 24,000 rpm for 3 h at 4 °C. The pelleted virions were suspended in 0.15M NaCl and 20 mM HEPES (pH 6.6), and LiDS and proteinase K were added to 5% and 400 μg/ml, respectively, After incubation at 42 °C for 10 min, the viral RNA was extracted with phenol followed by chloroform:isoamylalcohol (19:1). Viral RNA was ethanol-precipitated and the pellet was washed with 70% ethanol, dried under vacuum, and resuspended in water. Alternatively, 10^7^ 17Cl-1 cells were infected with virus, incubated for 13 h at 30 °C to 33 °C in 7.5% CO_2_. The poly(A)-containing RNA was then isolated from the infected cells using oligo-dT_25_ Dynabeads as described previously [62].

### RT-PCR and sequencing.

The entire replicase gene-coding region (ORF1a and ORF1b) was sequenced for eight *ts* mutant and revertant pairs. To do this, we used a set of 121 synthetic oligonucleotides that are complementary to sequences spaced at approximately 350 nucleotide intervals along the positive- and negative-strand copies of the viral RNA (sequences available on request). Five oligonucleotides, P17, P31, P46, P61, and P65, were used to prime the RT of viral RNA with Superscript II RT (Invitrogen, Carlsbad, California, United States). The reaction mix (20 μl), which contained, in addition to pre-supplied buffer, 35 ng of primer, 10–100 ng of viral RNA, 1 mM dNTPs, 10 mM DTT, 25 U of RNAguard (Amersham, Little Chalfont, United Kingdom), and 200 U of reverse transcriptase, was incubated at 42 °C for 60 min and then at 94 °C for 2 min. The five cDNA templates were then amplified using eight primer pairs, P1/P16, P2/P22, P3/P30, P4/P38, P5/P45, P6/P53, P7/P60, and P8/P64, and thermostable, recombinant *Taq* DNA polymerase. The reaction mix (100 μl), which contained, in addition to pre-supplied buffer, 70 ng of primer pair, 1 μl of RT reaction product, 200 μM dNTPs, 2 mM MgCl_2_, and 2.5 U of DNA polymerase, was incubated at 94 °C for 1 min, then 94 °C for 20 s, 50 °C for 20 s, 68 °C for 3 min, for a total of 35 cycles and a final 10-min extension at 68 °C. The PCR reaction products were purified by ethanol precipitation using ammonium acetate. Finally, sequence analysis was done using primers P1–P121 and standard cycle sequencing methods. Sequencing reaction mixes (10 μl), which contained 70 ng of primer, 100 ng of PCR product, and 3 μl of cycle sequencing mix (BigDye Terminator v.3.1, Applied Biosystems, Foster City, California, United States), were incubated at 96 °C for 10 s, 50 °C for 5 s, and 60 °C for 4 min, for a total of 25 cycles. The reaction products were purified by retention on a size exclusion membrane (Montage™ SEQ_96_, Millipore, Billerica, Massachusetts, United States) as described by the manufacturer; eluted and analyzed with an ABI 310 Prism Genetic Analyzer. Computer-assisted analysis of sequence data was done using the Lasergene bio-computing software (DNASTAR).

## Supporting Information

Figure S1 Plaque Morphology of Alb *ts*17 Revertants The plaque morphologies of Alb *ts*17_L_ and Alb *ts*17_S_ are illustrated. Alb *ts*17 had a reversion (back mutation) frequency of 2 × 10^−6^ and there was a mixture of large and small plaques at 40 °C. The virus from the small and large plaques produced progeny that formed uniformly small or large plaques at 40 °C, respectively. At 30 °C, both 17R_L_ and 17R_S_ produced plaques of equal diameter and Alb 17R_L_ produced the same size plaques at 40 °C as the parental or wt MHV-A59.
(1.7 MB PPT)

Table S1 Phenotypic Analysis of MHV-A59 *ts* Mutants (31 KB DOC)

## Abbreviations

CH: cycloheximide
CI: complementation index
EOP: efficiency of plating
hpi: hour post-infection
ORF: open reading frame
pfu: plaque-forming unit
TRS: transcription regulating sequence
ts: temperature-sensitive
wt: wild-type
