# Correction: Functional and Genetic Analysis of Coronavirus Replicase-Transcriptase Proteins

**DOI:** 10.1371/journal.ppat.0020017

**Published:** 2006-02-24

**Authors:** Stanley G Sawicki, Dorothea L Sawicki, Diane Younker, Yvonne Meyer, Volker Thiel, Helen Stokes, Stuart G Siddell

In *PLoS Pathogens,* vol 1, issue 4, DOI: 10.1371/journal.ppat.0010039

In the Results section, under the heading “Identification of Mutations Responsible for the *ts* Mutant Phenotype”:

The reversion of nucleotide 19288 in the Alb17Rs virus results in the substitution of Tyr with Ser, and not with Arg as originally stated.

Also in the Results section, under the heading “Phenotypes of the MHV-A59 *ts* Mutants”:

Virus-infected cells were labeled in the presence of 100 μg per ml of cycloheximide, and not 10 μg per ml as originally stated.

