# Supplementary material for: Functional and Genetic Analysis of Coronavirus Replicase-Transcriptase Proteins
Source: PLoS Pathog. 2005 Dec 9;1(4):e39. doi: 10.1371/journal.ppat.0010039 (PMC1298938; doi:10.1371/journal.ppat.0010039)
Supplement: Supplementary file 1 [file ppat.0010039.st001.doc]

# Supporting information

# Sawicki et al.

# Table S1. Phenotypic analysis of MHV-A59 *ts* mutants

| Virus |   ^3^H-uridine incorporation into viral RNA at ≥ 39˚C | Plaque formation at 37˚C* | EOP  pfu/ml  39.5˚C/30˚C |
| --- | --- | --- | --- |
| MHV-A59 | + | + | 10^0^ |
| Alb *ts*6 | - | - | 2 x 10^-5^ |
| Alb *ts*16 | - | - | 6 x 10^-6^ |
| Alb *ts*17 | - | - | 2 x 10^-6^ |
| Alb *ts*22 | - | -/+ | 2 x 10^-5^ |
| LA *ts*6 | - | - | 2 x 10^-8^ |
| Wü *ts*18 | - | + | 5 x 10^-7^ |
| Wü *ts*36 | - | + | 2 x 10^-5^ |
| Wü *ts*38 | - | + | 3 x 10^-4^ |
| Alb *ts*2 | - | - | 6 x 10^-6^ |
| Alb *ts*8 | - | - | 2 x 10^-5^ |
| Alb *ts*9 | - | - | 2 x 10^-5^ |
| Alb *ts*19 | - | - | <10^-9^ |
| LA *ts*3 | - | + | <10^-9^ |
| LA *ts*9 | - | + | 3 x 10^-5^ |
| NC *ts*2 | - | + | 4 x 10^-5^ |
| NC *ts*3 | - | + | 2 x 10^-6^ |
| Ut *ts*88 | - | nd | 3 x 10^-6^ |
| Ut *ts*145 | - | nd | 3 x 10^-5^ |
| Ut *ts*329 | - | nd | 10^-5^ |

* see text

# nd, not determined
